# Role of Delta/Notch-like EGF-related receptor in blood glucose homeostasis

**DOI:** 10.3389/fendo.2023.1161085

**Published:** 2023-05-08

**Authors:** Nelmari Ruiz-Otero, Rejji Kuruvilla

**Affiliations:** ^1^ Division of Endocrinology, Diabetes & Metabolism, Department of Medicine, Johns Hopkins University School of Medicine, Baltimore, MD, United States; ^2^ Department of Biology, Johns Hopkins University, Baltimore, MD, United States

**Keywords:** DNER, β-cell, pancreatic islets, cell-adhesion, N-CAM

## Abstract

Cell-cell interactions are necessary for optimal endocrine functions in the pancreas. β-cells, characterized by the expression and secretion of the hormone insulin, are a major constituent of functional micro-organs in the pancreas known as islets of Langerhans. Cell-cell contacts between β-cells are required to regulate insulin production and glucose-stimulated insulin secretion, which are key determinants of blood glucose homeostasis. Contact-dependent interactions between β-cells are mediated by gap junctions and cell adhesion molecules such as E-cadherin and N-CAM. Recent genome-wide studies have implicated Delta/Notch-like EGF-related receptor *(Dner)* as a potential susceptibility locus for Type 2 Diabetes in humans. DNER is a transmembrane protein and a proposed Notch ligand. DNER has been implicated in neuron-glia development and cell-cell interactions. Studies herein demonstrate that DNER is expressed in β-cells with an onset during early postnatal life and sustained throughout adulthood in mice. DNER loss in adult β-cells in mice (β-Dner cKO mice) disrupted islet architecture and decreased the expression of N-CAM and E-cadherin. β-Dner cKO mice also exhibited impaired glucose tolerance, defects in glucose- and KCl-induced insulin secretion, and decreased insulin sensitivity. Together, these studies suggest that DNER plays a crucial role in mediating islet cell-cell interactions and glucose homeostasis.

## Introduction

Novel genomic and proteomic analyses have allowed researchers to identify hundreds of candidates of genes involved in glucose homeostasis (1–4). However, understanding the molecular role and cellular expression, regulation, and overall function of identified genes is crucial to properly diagnose and treat diseases leading to glucose homeostasis defects, such as diabetes. Genome-wide studies in two independent populations have implicated Delta/Notch-like EGF-related receptor (*DNER*) as a potential susceptibility loci for Type 2 diabetes in humans (5, 6). DNER is a single-pass transmembrane protein with ten EGF-like repeats and a short cytoplasmic tail (7). Interestingly, the sequence and protein structure of DNER shows similarities with conserved Notch ligands, suggesting a role for DNER in Notch signaling (8, 9). DNER contains ten EGF-like domains, which are often implicated in protein-protein interactions, often at the plasma membrane where DNER is localized (7, 9–12). Moreover, previous studies have characterized a crucial role for DNER in mediating neuron-glia interactions (8, 13). DNER was first identified in the olfactory bulb, hippocampus and Purkinje cells in the cerebellum (7, 9). Studies in mice suggest that DNER induces the morphological differentiation of Bergmann glia, which are polarized astrocytes located in the cerebellar cortex (8). Mice with global deletion of DNER showed delayed maturation of the cerebellum, motor coordination deficits, and impaired glutamate removal from synapses (14). Importantly, DNER-Notch signaling is critical in the development and maturation of the central nervous system (8, 11, 13–15).

While DNER expression was initially believed to be restricted to the nervous system, studies have found that DNER is expressed in pancreatic tissues (16). Specifically, DNER expression was observed in mature β-cells using RNA-sequencing analysis (17). The onset of DNER expression in β-cells coincides with the period of β-cell maturation, when β-cells begin to coordinate their insulin release in response to glucose (17). Notably, β-cell maturation is characterized by increases in junctional protein content, where adhesive proteins and secretory machinery begin to assemble at the cell periphery for proper β-cell function (18–20). Furthermore, DNER was identified to be up-regulated upon glucose and cAMP stimulation in an insulin secreting cell line (3). Together, these findings suggest a potential role for DNER in blood glucose homeostasis. Here, we describe studies examining DNER function in the pancreatic β-cells and its role in blood glucose homeostasis in mice.

## Materials and methods

### Animals

All procedures relating to animal care and treatment conformed to Johns Hopkins University Animal Care and Use Committee (ACUC) and NIH guidelines. Animals were under direct supervision of the staff veterinarian and were monitored daily by Animal Care Staff. In addition to the sterile environment and weekly cage changes, mice were kept in a temperature-controlled room at 22°C and in a 12-hour light/12-hour dark cycle. Mice were maintained on a C57BL/6 background, or mixed C57BL/6J and 129P, or C57BL/6J and FVB backgrounds. Both sexes were used for analyses.

Dnertm3a KO first allele (C57BL/6NTac-Dnertm3a(EUCOMM)Hmgu/Ieg) mice were purchased from EMMA Repository (strain ID: EM:08389). Dnertm3a mice were crossed to a ROSA26-FLPe mouse (Jackson strain number: 003946) to knock out the LacZ and neo cassette to generate the conditional Dnertm3c allele (Dner-floxed) mice, which were then mated to *Pdx1-CreER* to generate *Pdx1-CreER; Dner^f/f^* (β-Dner cKO) mice.

Three-week old mice were weaned and introduced to control (TD.07570; Envigo) or tamoxifen diet (TD.130856; Envigo with 250 mg/kg diet) for 10 days with a 2-day break on standard chow after the fifth day. At eight weeks of age, control- and tamoxifen-fed animals were subjected to metabolic assays and pancreata were dissected for further morphological examination.

### Metabolic assays

#### Glucose tolerance test

Mice were housed individually and fasted overnight (16 hours) before glucose administration. Mice were administered 2g/kg of glucose by gavage feeding needle. Blood glucose levels were measured with a OneTouch Ultra 2 glucometer (Lifescan) before glucose administration and over a two-hour period. The area under the curve was determined for glucose tolerance for each animal using Graphpad Prism, version 9.

#### *In vivo* insulin secretion assay

Mice were housed individually and fasted overnight before glucose administration. Mice were given 3g/kg of glucose by gavage feeding needle. During a one-hour period, blood was collected in EDTA coated tubes and centrifuged. Insulin was measured from the plasma fraction collected using the Ultrasensitive Insulin ELISA (Crystal Chem, 90080).

#### Insulin sensitivity assay

Mice were individually housed with food overnight before being given an intraperitoneal (IP) insulin injection containing 0.75U/kg (Novolin-R, Novo Nordisk diluted in sodium chloride; Sigma-Aldrich). Blood glucose levels were assessed using a OneTouch Ultra 2 glucometer.

#### Islet isolation

Islets from adult animals were isolated as previously described (21). Pancreata were distended by injecting Collagenase P, from Clostridium histolyticum (Sigma-Aldrich 1124900200: 0.28mg/mL) diluted in Hanks’ Balanced Salt Solution (HBSS) (Gibco, 14175103) through the bile duct. Tissues were then incubated at 37°C, washed in 0.1% BSA in HBSS and subjected to discontinuous gradient using histopaque (6:5 Histopaque 1119: Histopaque 1077; Sigma-Aldrich 11191 and 10771) and HBSS. Islet layers were handpicked for further assays.

#### *Ex vivo* insulin secretion assay

Isolated islets were cultured in RPMI-1640 5%FBS 5U/L penicillin-streptomycin, (Gibco, 11875093) overnight to allow recovery. Islets were washed three times and were left pre-incubating for one hour in low glucose (2.8mM) Krebs Ringer Buffer (KRB). Groups of 5-10 islets were incubated in 500μL of either in low or high (16.7mM) glucose KRB for 30 minutes. After stimulation, the supernatant was collected and islets were lysed in acid ethanol (0.4N HCl, 75% EtOH) overnight. Islets were neutralized with Tris base (0.885M) and insulin was measured from the cell and supernatant fractions using the Ultrasensitive Insulin ELISA (Crystal Chem, 90080).

Total insulin content was measured by ELISA, normalized by the total amount of islets per sample, and averaged from at least two replicates per animal.

### Immunohistochemistry

#### Cryo-preserved tissues

Pancreata were dissected and fixed in 4% paraformaldehyde in PBS (PFA, Sigma) at 4°C overnight (16 hours). Tissues were cryo-protected in 30% sucrose in PBS for 24 hours and then equilibrated in 1:1 30%sucrose: OCT (Sakura Finetek) before embedding in OCT. Tissue was collected in 50μm thick sections.

Slides were washed in PBS, permeabilized in 1% Triton X-100 in PBS, and blocked for one hour at room temperature using 5% donkey serum 2%BSA in 0.5% Triton X-100 in PBS. Sections were then incubated for 24 hours at 4°C with primary antibodies (see **Table 1**). Following PBS washes, sections were incubated with corresponding secondary antibodies and DAPI for one hour at room temperature, washed, and mounted using Fluoromount Aqueous Mounting Medium.

**Table 1:** Reagents and resources.

Reagent or resource	Designation	Source or reference	Identifiers
Dner f/f mouse	*Dnertm1a(EUCOMM)Hmgu*	Helmholtz Zentrum Muenchen	MGI:4436436
Pdx1-CreER mouse	Tg(Pdx1-cre/Esr1*) #Dam/J; Pdx1- cre/Esr1*Dam	The Jackson Laboratory	MGI: 5008261
Pdx1-Cre mouse	B6.FVB-Tg(Pdx1-cre)6Tuv/Nci	The Jackson Laboratory	MGI: 5293639
Antibody	Guinea Pig anti insulin	Dako	Cat#: A0564
Antibody	mouse anti insulin	Sigma-Aldrich	Cat#: I2018
Antibody	goat anti DNER	R&D	Cat#: AF2254
Antibody	rabbit anti Somatostatin	Atlas antibodies	Cat# HPA019472
Antibody	Mouse anti glucagon	Abcam	Cat# ab10988
Antibody	rabbit anti N-CAM	Millipore	Cat#: AB5032
Antibody	rat anti E-cadherin	Invitrogen	Cat#: 13-1900
Antibody	rabbit anti Pdx1	Abcam	Cat#: ab47267
Reagent	Alexa Fluor™ 546 Phalloidin	Invitrogen	Cat#: A22283

The asterisk (*) represents the tamoxifen-inducible form of a cre recombinase/estrogen receptor fusion protein. The pound symbol (#) is used when no line is specified and/or lines are pooled (from Jackson laboratory website).

#### Paraffin embedded tissues

Pancreata were fixed in Bouin’s solution (Sigma-Aldrich, HT10132) at room temperature for four hours and left in 70% ethanol overnight. The pancreata were later dehydrated using increasing concentrations of ethanol, then incubated in a series of xylene and warm paraffin washes before being embedded in paraffin. Tissues were collected in 6μm thick sections.

Slides were washed in xylene and decreasing concentrations of ethanol to rehydrate. Then slides were washed with water and incubated in hot sodium citrate solution for antigen retrieval for 10 minutes. After cooling, slides were washed in PBS, incubated with glycine (0.1M), washed, and blocked for one hour on 5% goat serum 2%BSA in 0.5% Triton X-100 in PBS. Sections were then incubated overnight at 4°C with primary antibodies. Following PBS washes, sections were incubated with secondary antibodies and DAPI for one hour at room temperature, washed and mounted using Fluoromount Aqueous Mounting Medium.

### Imaging and image processing

Z-stacks were obtained using Zeiss LSM 700 microscopes equipped with 405, 488, 555, and 633 lasers. Images were processed using FIJI software. To measure the fluorescence intensity, an outline of the islet was made using the Fiji software to create the ROI. The area and integrated density of the desired channel was measured in each ROI. A second area was selected to serve as background and the mean grey value was measured. The corrected fluorescence intensity was calculated as: integrated density – (Islet area* mean grey fluorescence background readings).

### qRT-PCR

RNA was isolated from freshly isolated islets using Trizol Reagent (Invitrogen, 15596018) phenol-chloroform extraction and ethanol precipitation. RNA was then reverse transcribed using SuperScript IV First-Strand Synthesis System (Invitrogen, 18091050). Quantitative RT PCR was performed using Power SYBR Green PCR Master Mix (Applied Biosystems, 4367659) and the primer sets listed on **Table 2** on StepOnePlus Real-Time PCR System (ThermoFisher). Using 18S as endogenous control for normalization, the fold change was calculated using the 2^(-ΔΔCt)^ method.

**Table 2:** Primers used for qRT-PCR.

Gene symbol (Protein)	Forward	Reverse
18S	5’-CGCCGCTAGAGGTGAAATTC-3’	5’-TTGGCAAATGCTTTCGCTC-3’
*Dner*	5’-TGCCAGGACCAGTACATTGG-3’	5’-GCAAGTGAAATTGCTCCCATCC-3’
(DNER)		
*Ins1*	5’-CACTTCCTACCCCTGCTGG-3’	5’-ACCACAAAGATGCTGTTTGACA-3’
(Insulin)		
*Ins2*	5’-TGTCAAGCAGCACCTTTGTG-3’	5’-ACATGGGTGTGTAGAAGAAGCC-3’
(Insulin)		
*Gcg*	5’-ACC TGG ACT CCC GCC GTG CCC A-3’	5’-TCG CCT TCC TCG GCC TTT CAC CAG CC-3’
(Glucagon)		
*Sst*	5’-ACCGGGAAACAGGAACTGG-3’	5’-TTGCTGGGTTCGAGTTGGC-3’
(Somatostatin)		
*Ppy*	5’-CAGGCGACTATGCGACACC-3’	5’-CAGGGAATCAAGCCAACTGG-3’
(Pancreatic polypeptide)		
*Iapp*	5’-CCACTTGAGAGCTACACCTGT-3’	5’-GAACCAAAAAGTTTGCCAGGC-3’
(Islet amyloid polypeptide)		
*Ncam1*	5’-ACCACCGTCACCACTAACTCT-3’	5’-TGGGGCAATACTGGAGGTCA-3’
(N-CAM)		
*Cdh1*	5’-CAGGTCTCCTCATGGCTTTGC-3’	5’-CTTCCGAAAAGAAGGCTGTCC-3’
(E-cadherin)		
*Gjd2*	5’-ATGGGGGAATGGACCATCTTG-3’	5’-TCATCATCGTACACCGTCTCC-3’
(Connexin 36)		
*Pdx1*	5’-CCCCAGTTTACAAGCTCGCT-3’	5’-CTCGGTTCCATTCGGGAAAGG-3’
(Pdx1)		

### Quantifications and statistical analysis

Sample sizes were calculated based on power analysis and similar to those reported in previous publications (22–24). All analyses were performed in a semi-blinded manner, such that the investigator was not aware of the genotypes of each specific sample. All Student’s t-tests were performed assuming Gaussian distribution, two- tailed, unpaired, and with a confidence interval of 95%, except for normalized data (qRT-PCR and insulin sensitivity), which were done using a One sample t-test. Statistical analyses were based on at least 3 independent experiments and described in the figure legends.

## Results

### DNER expression in the pancreas

**Figure 1:**
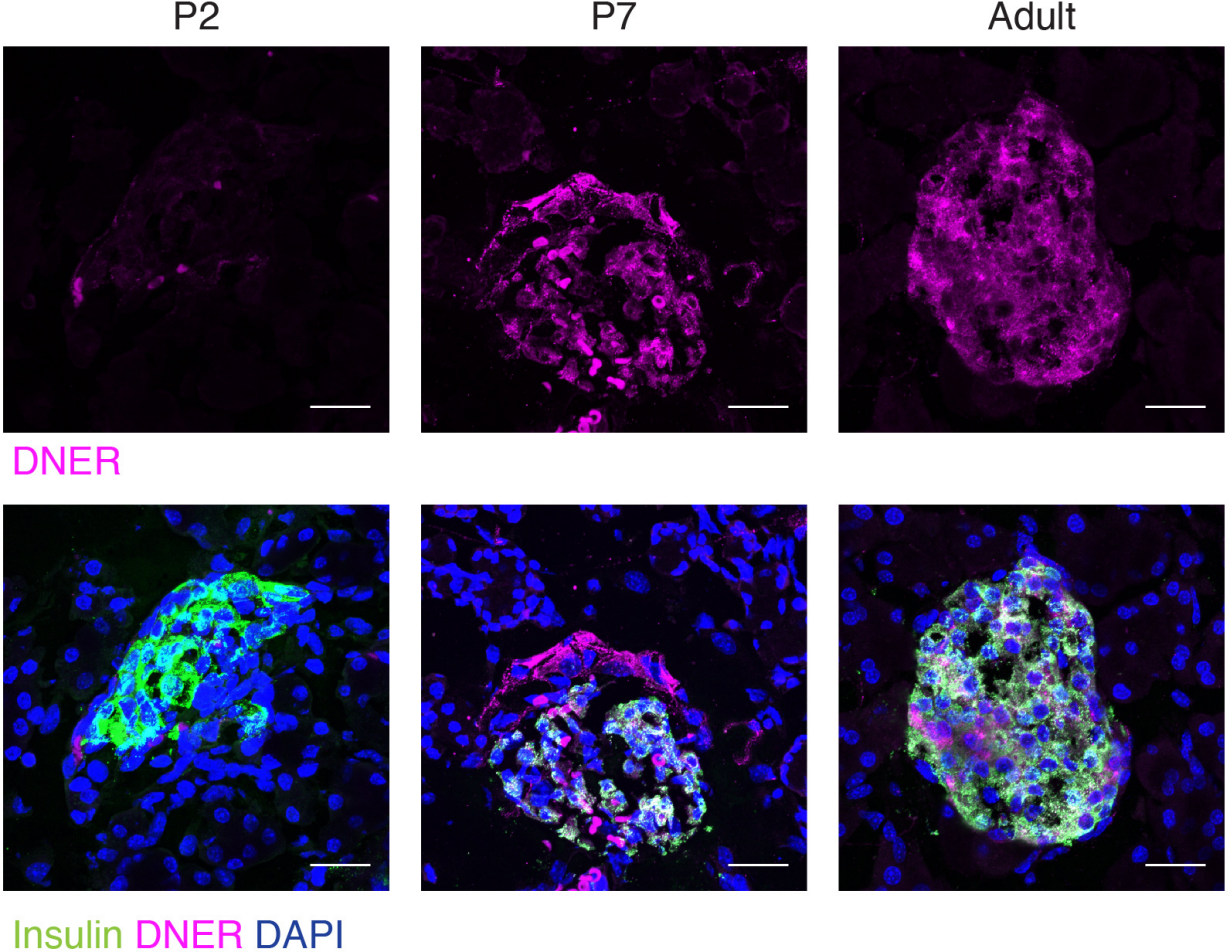
DNER expression in β-cells corresponds to the onset of β-cell maturation. Immunohistochemistry in wild-type tissues shows DNER (magenta) expression co-localizing with insulin (green) after P7. Scale bars: 25μm. Representative images from 3 independent experiments.

To define when and where DNER was expressed in the pancreas, we performed immunohistochemistry using an anti-DNER antibody in mice. DNER protein expression was mostly absent from the islets at perinatal periods, but after postnatal day seven (P7) and throughout adulthood, it was found mainly in insulin-producing β-cells with some limited expression in non-insulin expressing islet cells (**Figure 1**). Importantly, adult islets also show co-expression of DNER with glucagon (**Supplemental Figure 1**) These findings, together with recent evidence of transcriptional expression of DNER in mature β-cells (17) and its regulation by glucose in an insulin-producing cell line (3) suggested that DNER may play a functional role in mature β-cells.

### Functional characterization of mice lacking DNER in β-cells

To understand the role of DNER in mature β-cells, *Pdx1-CreER* (25) expressing mice were crossed with DNER floxed allele-containing mice (*Dner^f/f^*) (EUCOMM) (**Supplemental Figure 2A**). In the mature pancreas, Pdx1 is primarily expressed by the β-cells, although a small proportion of α-, PP- and δ-cells express Pdx1 (26, 27). Juvenile *Pdx1-CreER; Dner^f/f^* mice (P21) were introduced to either a control (TD.07570; Envigo) or tamoxifen diet (TD.130856; Envigo; 250 mg of tamoxifen/kg diet) for ten days to generate control or β-Dner cKO mice respectively. *Pdx1-CreER; Dner^f/f^* animals were born at expected Mendelian frequencies (from a *Pdx1-CreER; Dner^f/f^* to *Dner ^f/f^* cross 52.38% of the progeny was *Dner^f/f^* ^and^ 47.62% were *Pdx1-CreER; Dner^f/f^* mice; N= 6 litters and 42 animals), they did not display gross anatomical or morphological abnormalities and survived to adulthood. DNER loss in islets was confirmed using immunohistochemistry (**Supplemental Figure 2B**). Quantitative Real Time Polymerase Chain Reaction (qRT-PCR) analysis from isolated islets also showed a decrease in DNER transcripts (73% decrease) in β-Dner cKO islets relative to control islets (**Supplemental Figure 2C**), indicating significant deletion of DNER following induction with tamoxifen.

**Figure 2:**
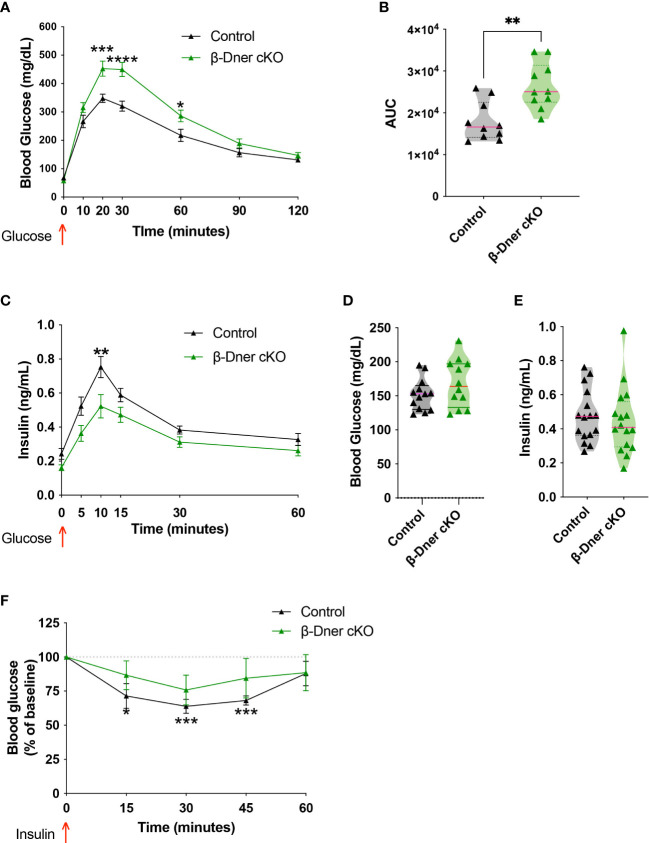
Loss of DNER from β-cells results in impaired glucose tolerance, decreased insulin secretion and loss of insulin sensitivity *in vivo.* **(A)** Glucose tolerance test (Means ± SEM for N = 10 animals per group: 2-way ANOVA *p < 0.05, ***p < 0.001, ****p < 0.0001). **(B)** Area under the curve (AUC) for glucose tolerance (t-test, **p<0.01). **(C)** Glucose-stimulated insulin secretion *in vivo* (Means ± SEM for N= 17 animals per group: 2-way ANOVA **p < 0.01). **(D, E)** Fed *ad libidum* levels of glucose and circulating insulin **(F)** Insulin sensitivity test (Means ± SEM for N= 6 controls and 7 β-Dner cKO animals: One sample t-test relative to baseline: *p < 0.05, ***p< 0.001).

To assess whether DNER plays a role in blood glucose homeostasis, glucose tolerance tests were performed in β-Dner cKO mice and litter-mate controls. β-Dner cKO animals exhibited impaired glucose tolerance at eight-weeks of age **(** **Figures 2A, B**). Defects in glucose tolerance typically arise due to impaired insulin secretion, lack of insulin sensitivity or a combination of both, although insulin-independent mechanisms may also influence glucose physiology (28, 29). To first determine whether insulin secretion was affected in mice lacking DNER in β-cells, a glucose-stimulated insulin secretion (GSIS) assay was performed *in vivo*. Adult β-Dner cKO mice had lower concentrations of circulating insulin after glucose stimulus (**Figure 2C**). However, levels of blood glucose and circulating insulin when fed *ad libitum* where similar in β-Dner cKO and littermate controls (**Figures 2D, E**). In addition, β-Dner cKO animals responded poorly to exogenously administered insulin, showing no significant decreases in glucose levels (**Figure 2F**). The insulin sensitivity defects were surprising given that DNER depletion in Pdx1-expressing cells is not expected to directly affect insulin-responsive tissues such as the liver or adipose tissue. Insulin resistance often arises from obesity, but female β-Dner cKO mice had similar body weight to controls, and male β-Dner cKO mice had decreased body weight than controls (**Supplemental Figure 3A**). However, males had a similar trend to glucose intolerance as females, although not statistically significant (**Supplemental Figures 3B, C**). Markedly, *in vivo* insulin secretion is significantly dampened in females but not in males, although a trend to a modest decreased insulin secretion is also present (**Supplemental Figures 3D, E**). Together, these results suggest a novel role for DNER in regulating blood glucose homeostasis.

**Figure 3:**
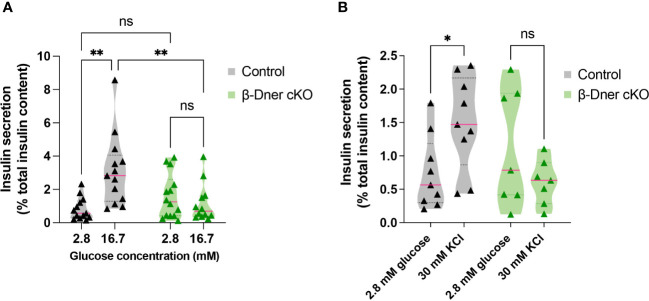
β-Dner cKO islets have intrinsic insulin secretion deficits. **(A)** Glucose-stimulated insulin secretion in isolated islets (Means ± SEM for islets from N= 10-12 animals per group: 2-way ANOVA **p < 0.01, ns p>0.05). **(B)** Depolarization-induced insulin secretion using KCl in isolated islets (Means ± SEM for islets from N= 7-9 animals per group: Two-way ANOVA *p < 0.05). ns or not significant: p>0.05.

To determine if the insulin secretion defects observed in β-Dner cKO mice were intrinsic to the islets, we performed glucose-stimulated insulin secretion assays using isolated islets from β-Dner cKO or control mice. As expected, a high concentration of glucose (16.7mM) elicited enhanced insulin secretion respective to basal glucose levels (2.8mM) in control islets. However, islets isolated from β-Dner cKO mice showed no difference in insulin secretion between low and high glucose concentrations (**Figure 3A**). Regulation of glucose-stimulated insulin release can occur at multiple levels including glucose sensing, metabolism, β-cell depolarization and insulin granule mobilization and exocytosis (30–33). To assess which steps in this pathway contributed to the diminished insulin secretion in β-Dner cKO islets, we treated isolated islets with a known insulin secretagogue, potassium chloride (KCl). High extracellular K+ depolarizes the β-cell plasma membrane, which by-passes the need for glucose metabolism to promote insulin secretion. Specifically, elevated potassium chloride (KCl, 30 mM) treatment results in the opening of voltage-dependent Ca^2+^ channels to promote exocytosis of secretion-ready insulin granules (34). We found that β-Dner cKO islets did not secrete insulin in response to high KCl in contrast to controls (**Figure 3B**), suggesting secretory defects independent of glucose entry and metabolism.

### Lack of DNER leads to low insulin levels

**Figure 4:**
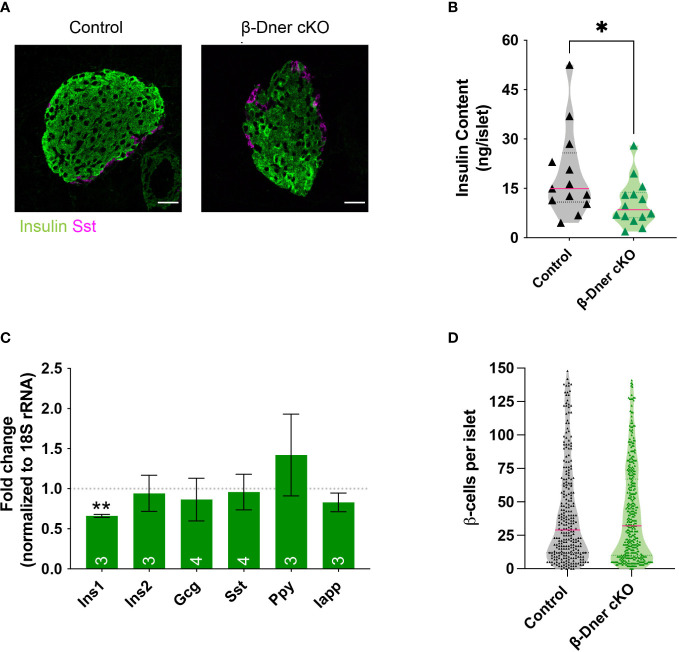
β-Dner cKO animals have lower insulin levels than littermate controls. **(A)** Immunohistochemistry of pancreatic islets stained for insulin (green) and somatostatin (magenta). Scale bars: 25μm. Representative images from 4 independent experiments. **(B)** Insulin protein content measured by ELISA (N=9-10 animals per group: t-test *p<0.05). **(C)** qRT-PCR analyses for endocrine markers, normalized by 18S and shown as relative to controls (Means ± SEM for islets from N= 3-4 animals per group: One sample t-test **p< 0.01). **(D)** β-cell number quantifications (N=4-5 animals per group; data shown as cells per islet).

Given the crucial role of islet morphology to its function, we decided to visualize control and DNER-depleted tissues using immunohistochemistry against islet hormones. We observed decreased insulin immunoreactivity (**Figure 4A**). Consistent with the decreased immunostaining, insulin protein content was reduced in β-Dner cKO islets (**Figure 4B**). Next, to determine whether insulin protein levels were decreased because of reduced RNA expression, we performed qRT-PCR on cDNA from isolated islets. Unlike humans, rodents contain two insulin coding genes: a functional transposon-mediated duplication *Ins1 and* the ancestral gene *Ins2*. The two transcripts are localized in different chromosomes and differ in some regulatory elements, including the exclusion of an intron in *Ins1*, and the regulation through FoxO1 of *Ins2* but not *Ins1* leading to differential modulation of their levels in certain conditions (35–37). Only *Ins1*, but not *Ins2* nor any other endocrine transcript tested were downregulated in DNER depleted islets (**Figure 4C**). The decrease observed in insulin was not due to loss of β-cells, as the number of β- cells per islet was unchanged versus control (**Figure 4D**). We also assessed α-cell number using glucagon immunostaining (**Supplemental Figures 4A, B**) and observed a decrease in glucagon+ α-cells. However, islet numbers were similar among control and β-Dner cKO tissues (**Supplemental Figure 4C**). Therefore, it is possible that DNER also acts regulating other islet endocrine cells. Taken together, these data indicate DNER in regulates insulin content in islets from adult mice.

### DNER mediates proteins involved in β-cell-cell interactions

**Figure 5:**
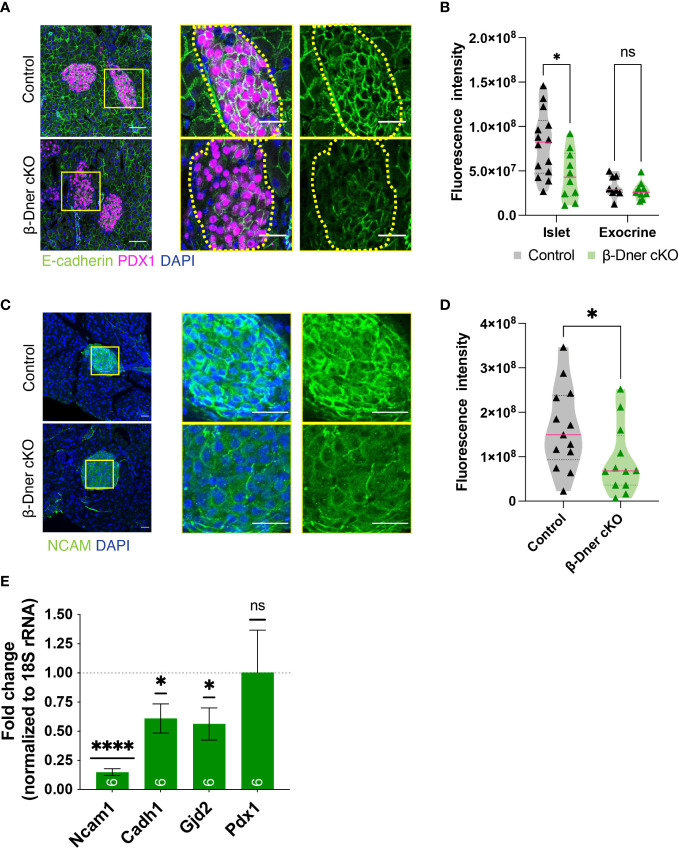
β-cell-cell contacts are disturbed in β-Dner cKO islets due to decreased expression of cell adhesion markers. **(A)** Immunohistochemistry for E-cadherin (green) and Pdx1 (magenta). Scale bars: 25μm. Representative images from 3 independent experiments. **(B)** Fluorescence intensity quantifications (arbitrary units) of E-cadherin immunohistochemistry in islets identified by Pdx1-positive immunohistochemistry and exocrine cells identified by DAPI-based nuclear morphology. (N= 3-4 animals per group, 2≥ islets per animal: t-test *p<0.05, ns p>0.05). **(C)** Immunohistochemistry for NCAM (green) shown in islets and innervating axons on the pancreas. Scale bars: 25μm. Representative images from 3 independent experiments. **(D)** Fluorescence intensity quantifications (arbitrary units) of NCAM immunohistochemistry. (N=3 animals per group, 3≥ islets per animal: t-test *p<0.05). **(E)** Levels of transcripts for NCAM (*Ncam1*), E-cadherin (*Cdh1*), Connexin 36 (*Gjd2*), and Pdx1 (*Pdx1*) measured by qRT-PCR, normalized by 18S and shown relative to controls (Means ± SEM for islets from N= 5-6 animals per group: One sample t-test ****p< 0.0001, * p<0.05). ns or not significant: p>0.05.

Since DNER regulates neuron-glia interactions in the CNS, we hypothesized that DNER might similarly regulate cell-cell interactions between islet cell types, either among β-cells and/or neighboring cells. To examine this possibility immunohistochemistry was used to visualize E-cadherin, a key mediator of β-cell-cell contacts. E-cadherin is expressed in multiple cell types in the pancreas, but plays an important role in promoting Ca^2+^-dependent intra-islet homophilic adhesions between β-cells (38–40). Interestingly, DNER deletion in β-Dner cKO mice resulted in decreased immunoreactivity of E-cadherin in islet cells (**Figures 5A, B**). This result was specific to islet cells, as exocrine pancreatic tissue near the islets did not show changes in E-cadherin levels (**Figures 5A, B**). These results provide evidence supporting of a role for DNER in regulating E-cadherin in pancreatic islets.

To further investigate the role of DNER in regulating β-cell-cell association, we assessed the levels of Neural Cell Adhesion Molecule (N-CAM), a transmembrane protein involved in Ca^2+^-independent cell-cell interactions and expressed in adult endocrine islets (41). N-CAM is necessary for segregation of cells within the mouse islet, specifically, the localization of α-cells to the islet periphery and the β-cells to the interior of the islets (41). Importantly, N-CAM also modulates exocytosis in both α- and β-cells (42). N-CAM immunoreactivity was largely reduced within β-Dner cKO islets (**Figures 5C, D**). We next performed qRT-PCR analyses to assess the levels of *Ncam1*, *Cadh1 and Gjd2*, which encode for N-CAM, E-cadherin and Connexin 36, respectively (**Figure 5E**). Connexin 36 is a gap junction protein expressed by β-cells and allows for synchronous Ca^2+^ waves to coordinate insulin secretion within islets (43). qRT-PCR analyses showed an 85% reduction of *Ncam1*, and approximately 40% reduction each for *Cadh1* and *Gjd2*. This was not a result of global suppression of transcription as Pdx1, also expressed by β-cells, showed no decrease in expression in β-Dner cKO islets. These results suggest that DNER loss results in the downregulation of genes involved in β-cell-cell interactions and adhesion.

### DNER regulates F-actin in β-cells

**Figure 6:**
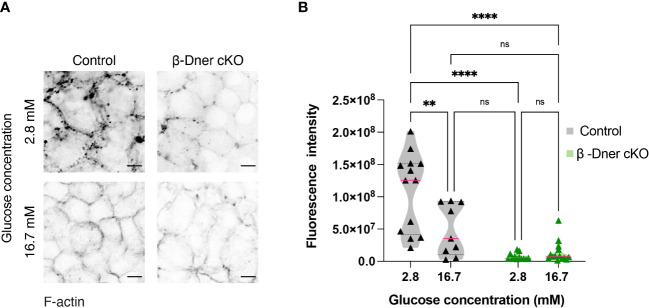
β-cells of β-Dner cKO have lower overall F-actin levels and impaired actin remodeling. **(A)** Immunohistochemistry of isolated islets incubated in 2.8mM or 16.7mM. Representative images from 3 independent experiments. **(B)** Fluorescence quantifications of isolated islets at different glucose concentrations (N=3 animals per group: 2-Way ANOVA **p<0.01, ****<0.0001). ns or not significant: p>0.05.

Upon DNER loss, islets showed decreased N-CAM and E-cadherin expression. N-CAM contains an intracellular domain, which is thought to bind the actin cytoskeleton and influence its remodeling (41, 42). Moreover, similar to our observation in β-Dner cKO mice, NCAM^-/-^ mice showed glucose intolerance and defective insulin secretion, which were attributed, at least in part, to impaired actin remodeling (42). Remodeling of cortical actin in response to a glucose stimulus allows docked insulin granules to access the plasma membrane and fuse and provides actin tracks for myosin-mediated transport of insulin granules that are stored deeper in cytoplasm (42, 44, 45). Additionally, re-arrangement of F-actin upon glucose exposure results in an increase in focal adhesions between β-cells, which are essential for optimal insulin secretion (46–48). Thus, we examined whether the glucose homeostasis defects in β-Dner cKO mice might be related to impaired actin remodeling, similar to that in NCAM^-/-^ mice. To determine if F-actin levels were altered in response to a glucose stimulus in β-Dner cKO β-cells, we stimulated islets with low (2.8mM) or high (16.7mM) glucose, and stained the islets with the F-actin binding dye, Phalloidin conjugated to 546-Alexa Fluor. As expected, in control islets, F-actin levels significantly decreased (an average of 45% reduction) after high glucose stimulation. In contrast, DNER depleted β-cells had significantly reduced F-actin levels at low glucose, which were significantly altered after the high glucose stimulation (**Figures 6A, B**). Together, these results suggest that DNER is required to maintain F-actin levels in β-cells, which is crucial for the regulated secretion of insulin granules in response to glucose.

## Discussion

Here, we have established that DNER is expressed within pancreatic β-cells, and that loss of DNER leads to glucose intolerance, decreased insulin secretion and insulin resistance in adult mice. Furthermore, loss of DNER resulted in decreased islet insulin content and diminished expression of genes critical for cell-cell interactions (*Ncam1, Cadh1 and Gjd2*). Together, these findings support a physiological role for DNER in regulating blood glucose homeostasis, likely *via* regulating β-cell-cell association.

Glucose intolerance arises from defects in glucose-stimulated insulin secretion from the pancreatic β-cells, dampened responses to insulin in insulin-sensitive tissues such as the liver, adipose tissues or skeletal muscles, or a combination of defects in both insulin secretion and sensitivity. Moreover, blood glucose uptake can be regulated by insulin-independent mechanisms, including a combination of autonomic and neuroendocrine factors, as well as a well-described involvement of the CNS in postprandial glucose regulation (49, 50). β-Dner cKO mice had impaired glucose-stimulated insulin secretion, which at least in part, may result from defective β-cell-cell communication and actin dynamics. Decreased insulin secretion in β-Dner cKO mice can be explained by dampened levels of islet insulin, and also by defects in the secretory capacity of β-cells. Our experiments in isolated islets showed intrinsic defects in glucose stimulated insulin release, and defects downstream of β-cell depolarization, as seen by the lack of response to KCl in mutant islets. Importantly, diminished insulin secretion in response to KCl indicates secretory defects independent of glucose entry and metabolism. Future studies will be required to determine whether DNER’s activity in the β-cells directly modulates insulin secretion, or whether the defects observed are an indirect consequence of DNER loss.

Depletion of DNER in β-cells resulted in insulin resistance. Insulin resistance often arises from hyperinsulinemia or obesity (4, 51, 52). Neither hyperinsulinemia (**Figures 2C, E**) nor increased body weight was observed in β-Dner cKO animals (**Supplemental Figures 2A, B**). It is possible that defects in insulin sensitivity could arise due to DNER ablation in some Pdx1-positive cells outside of the pancreas (53–55). Some studies using *Pdx1-Cre* or *Pdx1-CreER* animals crossed to genetic reporter lines indicated recombination in cells present in the medial preoptic area, raphe nucleus, inferior olivary nucleus, lateral hypothalamus, arcuate nucleus, and dorsomedial hypothalamus (53–55). The hypothalamus is a non-classical insulin-responsive brain region in which astrocytes promote glucose availability in the CNS and systemic glucose metabolism (56–59). Moreover, DNER loss in the CNS has previously been linked to defects in neuron- glia interactions, crucial for their development and function (8, 13). Therefore, loss of DNER expression in Pdx1-positive hypothalamic neurons could potentially contribute to defects in insulin sensitivity and glucose homeostasis in β-Dner cKO mice. Future studies using other pancreas-specific or hypothalamic-specific Cre lines are necessary to examine DNER regulation of insulin-sensitive tissues. Nevertheless, experiments performed in isolated islets lacking DNER *in vitro* showed insulin release defects demonstrating that the insulin secretory defect is at least in part intrinsic to the islet. Thus, these results demonstrate that DNER function is critical to the normal physiological function of pancreatic β-cells in regulating insulin secretion and blood glucose.

Surprisingly, β-cells from β-Dner cKO animals had reduced expression of N-CAM and E-cadherin, both at the protein and the transcript level. Furthermore, the transcript for *Gjd2*, which encodes for connexin 36 was also downregulated. Connexin 36 is the major gap junction component expressed in β-cells, and it’s loss leads to defects in calcium signaling and coordination of insulin release within islets (60–62). Therefore, we speculate that reduction of connexin 36 and consequently a dysregulation of calcium dynamics within the islets might be, at least in part, responsible for the insulin secretory defects observed in β-Dner cKO mice.

Our results showing DNER expression in postnatal β-cells as well as previous reports of its enrichment in mature β-cells in sequencing studies (17), indicate a role for DNER in the functionally mature β-cells. Importantly, maturation of β-cells correlates with increased expression and functional localization of connexins, secretory machinery, ion channels and other proteins localized in inter-cellular spaces, including N-CAM and E-cadherin (17–20, 63). Our results suggest that DNER is necessary for the expression, and perhaps, localization of N-CAM and E-cadherin in the plasma membrane in mature β-cells.

Prior studies demonstrated N-CAM was required for F-actin remodeling in islets upon glucose stimulation (42). Like β-Dner cKO animals, NCAM^-/-^ mice displayed defects in insulin secretion. Given the decreased N-CAM expression in DNER depleted β-cells, we investigated whether F-actin levels were affected in β-Dner cKO islets. In contrast to NCAM^-/-^ islets, where a denser submembrane actin network was observed even in high glucose concentrations (15mM or 30mM) (42), we observed decreased levels of F-actin in β-Dner cKO isolated islets, irrespective of glucose concentrations. Whether the reduced F-actin is due to defective actin assembly, decreased actin protein levels, or a combination of these factors requires further investigation. Upon glucose stimulation, cortical F-actin is remodeled to allow insulin granule access to the plasma membrane, provide myosin-mediated transport of insulin granules from the cytoplasm to the plasma membrane, and to form focal adhesions which provide a structural and molecular link between the extracellular matrix (ECM), the cytoskeleton and signaling molecules (44–48). Glucose stimulation promotes the formation of these contact sites downstream of Ca^2+^ entry. Furthermore, p-FAK and paxillin regulate the localization of SNAP-25 and syntaxin-1, which are crucial for insulin granule exocytosis (47, 48). If β-Dner cKO islets are unable to form focal adhesions and localize exocytic machinery, that could be a potential mechanism to link the loss of F-actin with the dampened insulin secretion. How DNER modulates the expression of adhesive molecules and regulates the actin cytoskeleton remains to be answered. Whether DNER physically interacts with adhesive proteins or if it directly modulates F-actin levels are open questions for future studies. Nonetheless, this study shows that DNER plays a novel role in maintaining β-cell-cell contacts and maintaining islet morphology. Specifically, our findings suggest that defects in insulin secretion arise, in part, from dysregulation of the actin cytoskeleton and decreased expression of adhesive markers in β-Dner cKO islets.

*DNER* alleles in humans have been correlated with insulin resistance phenotypes in Type 2 Diabetes patients (5, 6). However, the specific cell types expressing DNER and the molecular pathways influenced by DNER to elicit disease pathology were not evaluated. We anticipate that the mechanisms we have defined in this study will provide a foundation for future studies to fully elucidate the role of DNER in the healthy pancreas, and to assess whether DNER mutations or polymorphisms contribute to diabetes risk. Targeting of DNER or other cell-cell signaling pathways may represent a novel therapeutic strategy for diabetes patients.

## Data availability statement

The original contributions presented in the study are included in the article/**Supplementary Material**. Further inquiries can be directed to the corresponding author.

## Ethics statement

The animal study was reviewed and approved by Johns Hopkins University Animal Care and Use Committee (ACUC).

## Author contributions

NR-O contributed to study design, investigations, data analyses, and writing and editing the manuscript. RK contributed to writing/editing the manuscript and funding acquisition. All authors contributed to the article and approved the submitted version.

## Supplementary material

Supplementary Figure 1 DNER is expressed in endocrine α-cells. **(A)** Immunohistochemistry of adult wild-type pancreatic tissues shows DNER (red) and glucagon (green). Scale bars: 25μm. Representative images from 3 independent experiments.

Supplementary Figure 2 DNER deletion from adult β-cells in β-Dner cKO animals. **(A)** Schematic of *Dnerf/f* (Dnertm3c) mouse generation and β-Dner cKO from Dnertm3a (EUCOMM) [adapted from Groza et al. (64)]. **(B)** Immunohistochemistry of β-Dner cKO islets (outlined) shows reduced DNER (magenta) expression. Scale bars: 25μm. Representative images from 3 independent experiments. **(C)** qRT-PCR for DNER transcripts, normalized by 18S and shown as relative to controls (N=7 animals per group: One sample t-test ****p < 0.0001).

Supplementary Figure 3 β-Dner cKO animals are not obese. β-Dner cKO males and females have similar changes in glucose tolerance and insulin secretion. **(A)** Weight of mice at 8-weeks of age (N= 10-12 animals per group: t-test comparison per sex: *p<0.05). **(B)** and **(C)** Glucose tolerance test of males and females respectively (Means ± SEM for N = 5 animals per group: 2-way ANOVA *p < 0.05, *** p < 0.001, ****p < 0.0001). **(D)** and **(E)** Glucose-stimulated insulin secretion *in vivo of* males and females respectively (Means ± SEM for N= 7-10 animals per group: 2-way ANOVA **p < 0.01).

Supplementary Figure 4 β-Dner cKO have normal islet endocrine organization and decreased α-cell number **(A)** Immunohistochemistry of pancreatic islets stained for glucagon (green) and Pdx1 (magenta). Scale bars: 50μm. Representative images from 3 independent experiments. **(B)** α-cell number quantifications (N=4-5 animals per group; data shown as cells per islet, t-test **p<0.01). **(C)** Islet number per pancreatic section (N=4-5 animals per group). Pancreatic sections were quantified every 100μm. **(D)** Levels of transcripts measured by qRT-PCR, normalized by 18S and shown relative to controls (Means ± SEM for islets from N ≥ 2 animals per group).
