# Supplementary figures and images for: Role of Delta/Notch-like EGF-related receptor in blood glucose homeostasis
Source: Front Endocrinol (Lausanne). 2023 May 8;14:1161085. doi: 10.3389/fendo.2023.1161085 (PMC10200888; doi:10.3389/fendo.2023.1161085)

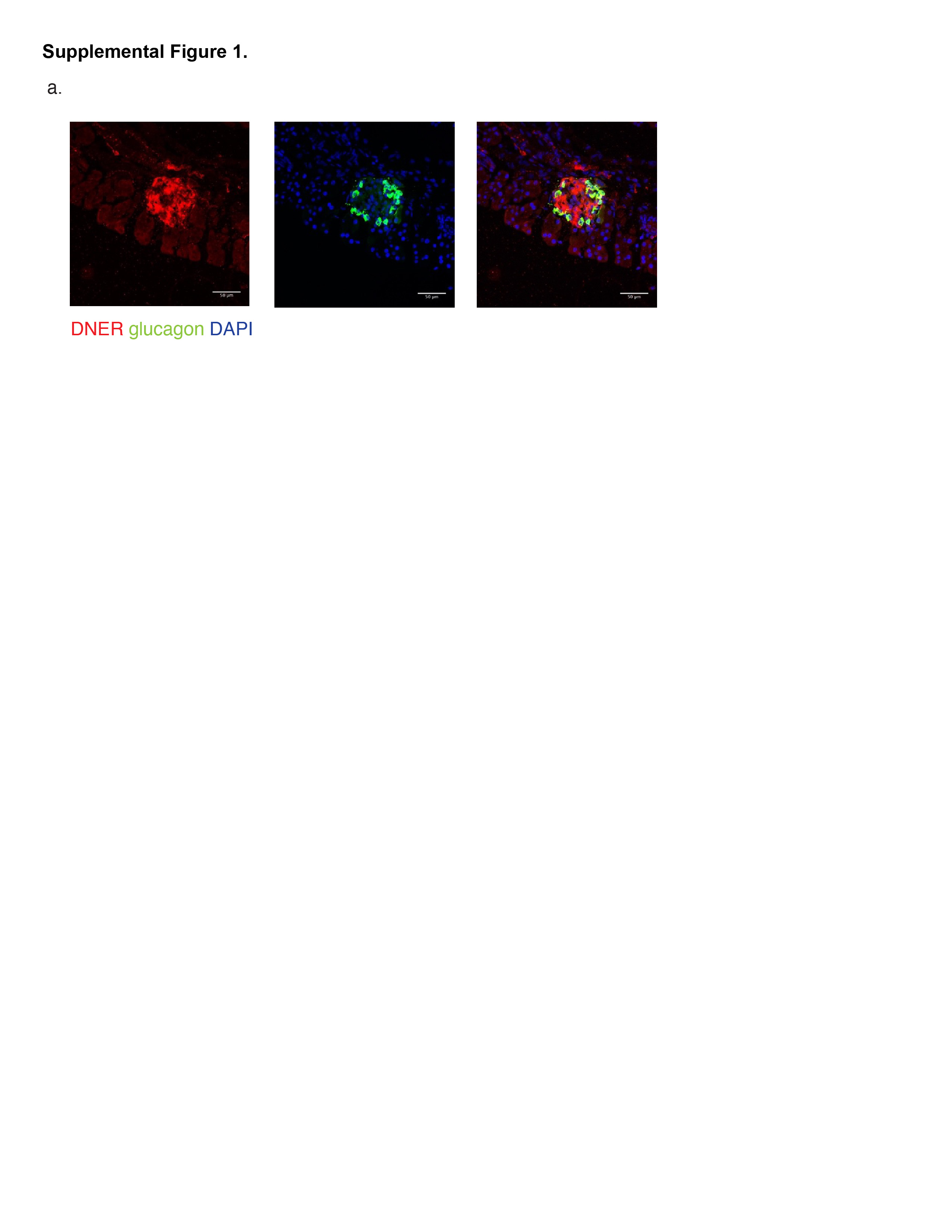

Supplement: Supplementary Figure 1 — DNER is expressed in endocrine α-cells. (A) Immunohistochemistry of adult wild-type pancreatic tissues shows DNER (red) and glucagon (green). Scale bars: 25μm. Representative images from 3 independent experiments. [file Image_1.jpeg]

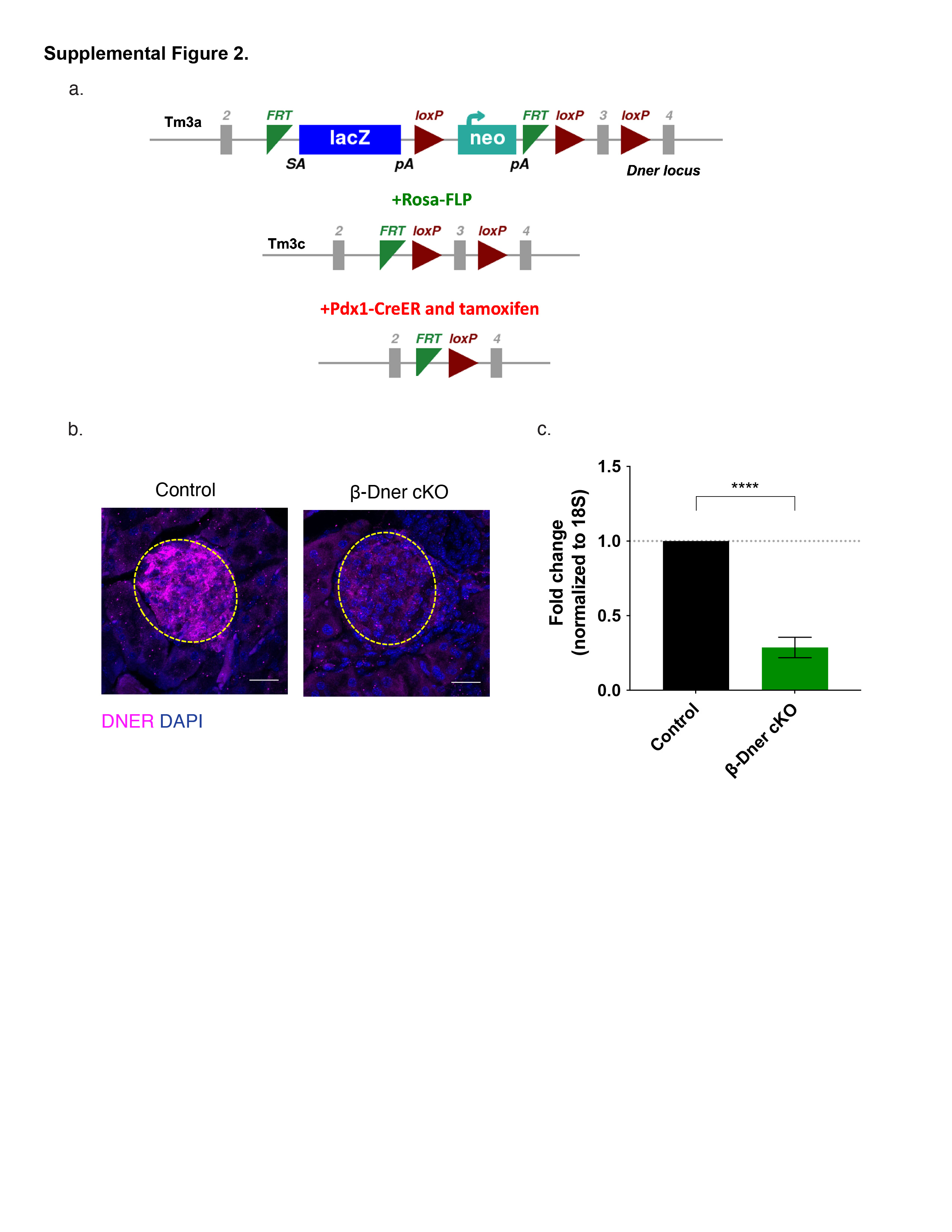

Supplement: Supplementary Figure 2 — DNER deletion from adult β-cells in β-Dner cKO animals. (A) Schematic of Dnerf/f (Dnertm3c) mouse generation and β-Dner cKO from Dnertm3a (EUCOMM) [adapted from Groza et al. (64)]. (B) Immunohistochemistry of β-Dner cKO islets (outlined) shows reduced DNER (magenta) expression. Scale bars: 25μm. Representative images from 3 independent experiments. (C) qRT-PCR for DNER transcripts, normalized by 18S and shown as relative to controls (N=7 animals per group: One sample t-test ****p < 0.0001). [file Image_2.jpeg]

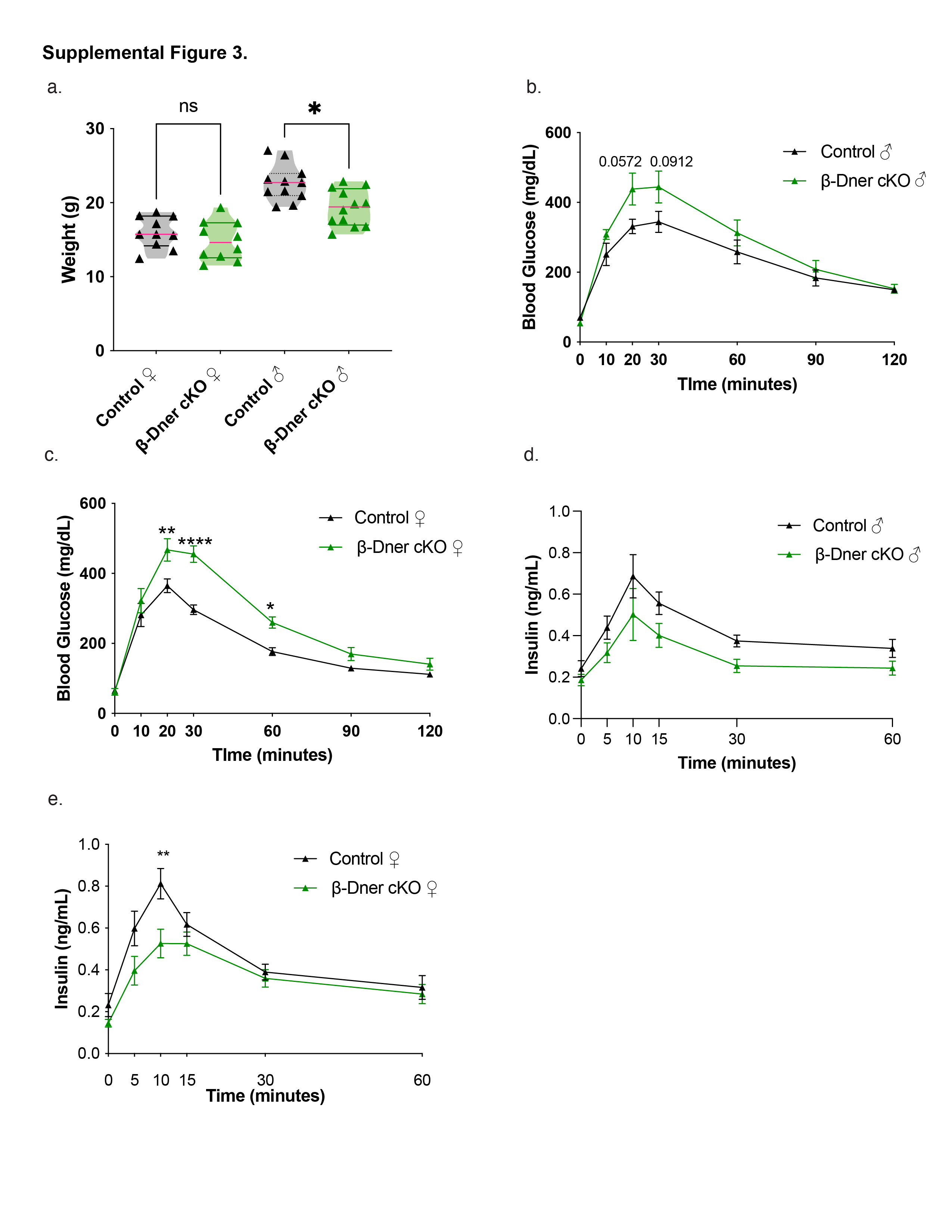

Supplement: Supplementary Figure 3 — β-Dner cKO animals are not obese. β-Dner cKO males and females have similar changes in glucose tolerance and insulin secretion. (A) Weight of mice at 8-weeks of age (N= 10-12 animals per group: t-test comparison per sex: *p<0.05). (B) and (C) Glucose tolerance test of males and females respectively (Means ± SEM for N = 5 animals per group: 2-way ANOVA *p < 0.05, *** p < 0.001, ****p < 0.0001). (D) and (E) Glucose-stimulated insulin secretion in vivo of males and females respectively (Means ± SEM for N= 7-10 animals per group: 2-way ANOVA **p < 0.01). [file Image_3.jpeg]

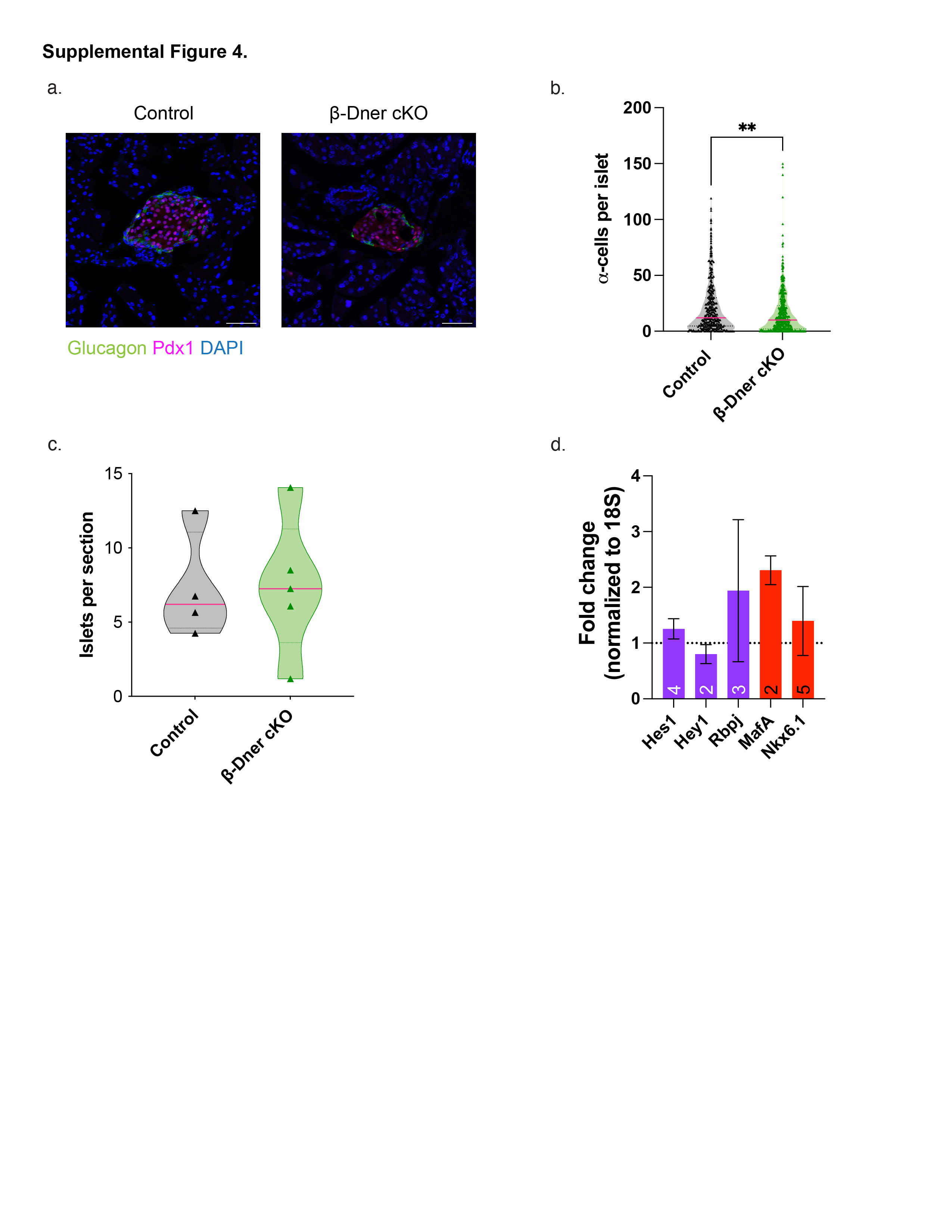

Supplement: Supplementary Figure 4 — β-Dner cKO have normal islet endocrine organization and decreased α-cell number (A) Immunohistochemistry of pancreatic islets stained for glucagon (green) and Pdx1 (magenta). Scale bars: 50μm. Representative images from 3 independent experiments. (B) α-cell number quantifications (N=4-5 animals per group; data shown as cells per islet, t-test **p<0.01). (C) Islet number per pancreatic section (N=4-5 animals per group). Pancreatic sections were quantified every 100μm. (D) Levels of transcripts measured by qRT-PCR, normalized by 18S and shown relative to controls (Means ± SEM for islets from N ≥ 2 animals per group). [file Image_4.jpeg]
